# Impacts of technology on children’s health: a systematic review

**DOI:** 10.1590/1984-0462/2023/41/2020504

**Published:** 2022-07-06

**Authors:** Raquel Cordeiro Ricci, Aline Souza Costa de Paulo, Alisson Kelvin Pereira Borges de Freitas, Isabela Crispim Ribeiro, Leonardo Siqueira Aprile Pires, Maria Eduarda Leite Facina, Milla Bitencourt Cabral, Natália Varreira Parduci, Rafaela Caldato Spegiorin, Sannye Sabrina González Bogado, Sergio Chociay, Talita Navarro Carachesti, Mônica Mussolini Larroque

**Affiliations:** a Universidade Federal de Mato Grosso do Sul, Três Lagoas, MS, Brazil.

**Keywords:** Internet access, Growth, Child development, Pediatrics, Technology, Acesso à internet, Crescimento, Desenvolvimento infantil, Pediatria, Tecnologia

## Abstract

**Objective:**

To identify the consequences of technology overuse in childhood.

**Data source:**

A systematic review was carried out in the electronic databases PubMed (National Library of Medicine of the National Institutes of Health) and BVS (Virtual Health Library), considering articles published from 2015 to 2020, in English, Portuguese and Spanish using the terms “Internet”, “Child” and “Growth and Development”.

**Data synthesis:**

554 articles were found and 8 were included in the analysis. The studies’ methodological quality was assessed by the Strobe and Consort criteria, being scored from 17 to 22 points. The articles showed positive and negative factors associated with the use of technology in childhood, although most texts emphasize the harmful aspects. Excessive use of internet, games and exposure to television are associated with intellectual deficits and mental health issues, but can also enable psychosocial development.

**Conclusions:**

Preventing the use of the internet is a utopic measure ever since society makes use of technologies. The internet is associated with benefits as well as with harms. It is important to optimize the use of internet and reduce risks with the participation of parents and caregivers as moderators, and training of health professionals to better guide them.

## RESUMO

**Objetivo:**

Identificar as consequências do uso excessivo da tecnologia na infância.

**Fontes de dados:**

Foi realizada uma revisão sistemática nas bases de dados eletrônicas PubMed (National Library of Medicine — National Institutes of Health) e Biblioteca Virtual em Saúde (BVS) com artigos publicados de 2015 a 2020, em inglês, português e espanhol, utilizando os termos *internet, child* e *growth and development*.

**Síntese dos dados:**

Foram localizados 554 artigos, resultando em oito artigos incluídos nesta pesquisa. Os estudos foram avaliados quanto à sua qualidade metodológica pelos critérios Strengthening the Reporting of Observational Studies in Epidemiology (Strobe) e Consolidated Standards of Reporting Trials (Consort) e receberam pontuações que variaram de 17 a 22 pontos. Os artigos evidenciaram que há fatores positivos e negativos associados ao uso de tecnologias na infância, embora a maioria dos textos ressalte seu aspecto prejudicial. O uso excessivo de internet, jogos e exposição à televisão ocasionaram alterações intelectuais e da saúde mental, porém também possibilitaram o desenvolvimento psicossocial.

**Conclusões:**

Impedir o uso da internet é uma medida utópica, visto que a sociedade faz uso de tecnologias. Considerando que a internet pode trazer benefícios, mas também malefícios, são importantes a otimização do uso e a redução dos riscos, como a participação dos pais e responsáveis como moderadores dessa utilização, além da atualização dos profissionais da saúde para melhor orientá-los.

## INTRODUCTION

Nowadays, information and communication technologies increasingly make up children’s daily routines. Data from the Brazilian Institute of Geography and Statistics (IBGE) state that, among Brazilian children aged 10 years and over, internet use rose from 69.8% in 2017 to 74.7% in 2018. Exchange of messages, voice and/or video calls and, finally, watching videos, such as series and movies, are the most frequent activities performed requiring internet services.^1^

Studies on digital technologies have been carried out in several fields, since the contents of activities on the internet may vary, reflecting the broad range of information available online. From this perspective, much has been questioned about the impacts of information and communication technologies on children’s physical and psychosocial development. In the cognitive sphere, the influence on sleep, memory, reading ability, concentration, the ability to communicate in person are commonly cited, in addition to anxiety symptoms when children are away from their cell phones.^2,3^

This construction of self-image by means of technological tools results in potentializing a phenomenon of modernity and the emergence of large cities: placing intimacy as the focus of spectacularization. Furthermore, intense consumption of content can cause anxiety, panic and even depression. In the case of children with previous mental health conditions and who require monitoring, these effects can be even more intense.^4^

With this in mind, the World Health Organization (WHO) published a series of recommendations to parents regarding the exposure of children of different age groups to digital technologies. Children under the age of 5 should not spend more than 60 minutes a day in passive activities in front of a smartphone, computer or TV screen. Children under 12 months of age should not spend even a minute in front of electronic devices. The goal is for boys and girls up to 5 years old to change electronics for physical activities or practices that involve interactions in the real world, such as reading and listening to stories with caregivers.^5^ These guidelines are part of the strategy for awareness on sedentary lifestyle and obesity by the Organization of United Nations (UN).

Thus, it is clear that this spectrum of influence can culminate or intensify various pathologies. Therefore, the aim of the study was to identify the positive and negative consequences of technology overuse in childhood.

## METHOD

The selection process and the development of this systematic review were based on the Preferred Reporting Items for Systematic Reviews and Meta-Analyses (Prisma) protocol.^6^ This review was registered with the International Prospective Registry of Systematic Reviews (Prospero), under number CRD42021248396.

The National Library of Medicine — National Institutes of Health (PubMed) and Virtual Health Library (VHL) electronic databases were searched from March to July 2020. The purpose was to systematically analyze original studies addressing information technologies and communication (Internet, social media, etc.) in child development based on a guiding question: what is the impact of information and communication technologies on childrens physical and psychosocial development?

The Medical Subject Headings (MeSH) was used to define the search term. Then, an exploratory investigation was carried out with the purpose of identifying keywords within the theme. The terms “internet”, “child” and “growth and development” were used, in English language, along with “AND”, to combine them. Additionally, the bibliographic references of articles selected were checked.

For the articles to be included, the following aspects were considered:

- Original articles.
- Studies conducted with children.
- Research regarding information and communication technologies (Internet, television, etc.) related to child development.
- Published from 2015 to 2020.
- Articles written in English, Portuguese and Spanish.

Studies carried out with adolescents, adults and the elderly, as well as theses, dissertations, monographs, duplicate studies and case studies were excluded.

The search and selection of articles took place at two different times. The articles were selected first by title and abstracts and, then, the full texts were accessed and evaluated.

Studies that met the eligibility criteria were fully analyzed by two independent researchers, whose evaluations were then compared to verify common points. In cases of uncertainty about the eligibility of the study, a third evaluator took part. Then, the data was extracted and input in predefined data tables.

The methodological quality of observational articles included was assessed according to the initiative Strengthening the Reporting of Observational Studies in Epidemiology (Strobe), based on various evaluation criteria for this type of studies. The maximum score is 22 points, which are distributed over several items: title and/or abstract (one item), introduction (two items), methodology (nine items), results (five items), discussion (four items), and funding (one item).^7,8^ All observational studies were evaluated, and each item, when present, added up to 1 point; then the sum was scored according to Table 1.

**Table 1:** Design of studies included in the systematic review (n=8).

Authors (year)	Desig	Sample size+age groups/parents	Study quality (score)^7, 9^
McNeill et al. (2019)^16^	Longitudinal	185 children aged 3–5 years. Australia	22^a^
Takeuchi et al. (2018)^3^	Cohort	507 children (cross-sectional=284 aged 5.7–18.4 years, and longitudinal=223 aged 8.4–21.3 years). Japan	21^a^
Folkvord et al. (2017)^15^	Randomized controlled trial	562 children. Netherlands (211 children aged 6–11 years) and Spain (351 children aged 6–12 years)	18^b^
Yu and Park (2017)^17^	Longitudinal	2,840 children with mean age of 9.86 ± 0.35 years. South Corea.	20^a^
Slater et al. (2017)^13^	Case control	80 girls aged 8–9 years. England.	20^a^
Takeuchi et al. (2016)^11^	Longitudinal and cross-sectional	429 children (cross-sectional=240 aged 5.7–18.4 years; longitudinal=189 aged 8.4–21.3 years). Japan	19^a^
Slater et al. (2016)^14^	Longitudinal	300 girls aged 6–9 years. Australia	17^a^
Takeuchi et al. (2015)^12^	Longitudinal and cross-sectional	1,071 children aged 5.6–18.4 years (prior to study=290; after study=235; cross-sectional=276; longitudinal: 216). Japan	20^a^

a based on Strengthening the Reporting of Observational Studies in Epidemiology (Strobe)^7^

b based on the Consolidated Standards of Reporting Trials (Consort) 2010.^9^

The methodological quality of the one randomized trial was based on the Consolidated Standards of Reporting Trials (Consort) strategy, which contains a checklist with 25 items, divided into: title and abstract (one item with two sub-items); introduction (one item with two sub-items); methods (five items) and a topic with information about randomization (five items); results (seven items); discussion (three items); and other information, such as registration, protocols and funding (three items).^9,10^ Each item, if met, equals 1 point, and they were all added up according to the analysis of the papers. The score of methodological quality of this randomized trial is shown in Table 1.

In order to synthesize the description of characteristics as main results and descriptive approach, the following information was extracted from each selected article: name of the main author, year of publication, country where the study was performed, design, sample size, type of technology evaluated, statistical variables, main results, and limitations.

## RESULTS

**Figure 1:**
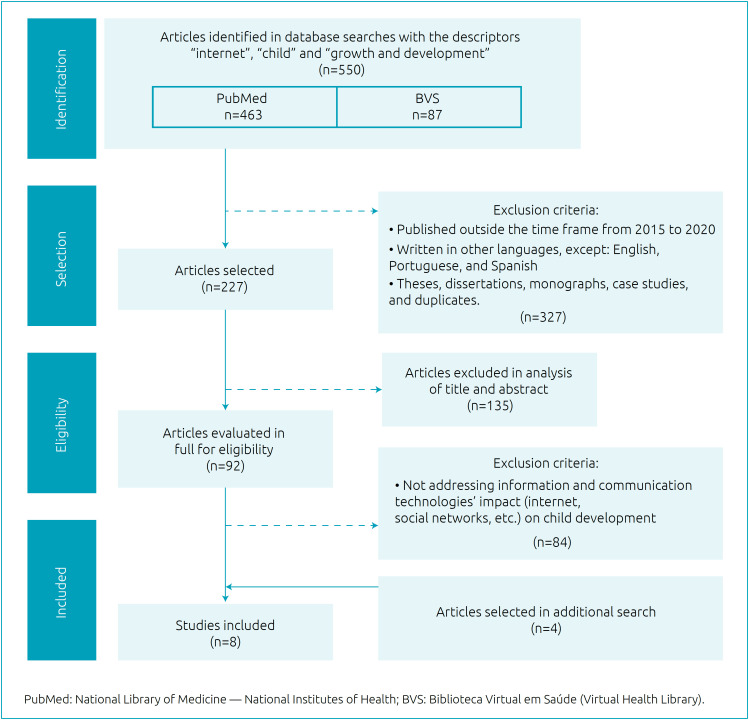
Flowchart of the selection process.

Source: adapted from Preferred Reporting Items for Systematic Reviews and Meta-Analyses (Prisma) statement.^9^

Searches on PubMed and VHL using the descriptors “internet”, “child” and “growth and development” retrieved 550 articles. After applying inclusion criteria, 221 studies were selected and, after reading the titles and abstracts, 125 were excluded. 92 articles were read in full and, per the inclusion criteria and a detailed analysis, four studies were selected. Four other articles were included after an additional search in the reference list of primarily selected articles; the studies should have the same inclusion criteria defined in the methodology. Thus, eight articles made up the sample. The flowchart is shown in Figure 1.

Most studies were epidemiological. Almost all of them were observational (n=7), and only one was an intervention study. The observational studies included were longitudinal and/or cross-sectional (n=5), case-control (n=1) and cohort studies (n=1). Only one experimental study was included, a randomized controlled trial (n=1), as shown in Table 1.

Their methodological quality was based on their scores (Table 1). Most studies were observational (n=7) and, therefore, were evaluated according to the Strobe criteria^7^. The score ranged from 17 to 22, and most articles reached 20 points (n=4), which is good methodological quality. The quality of the randomized trial with 18 points—according to the Consort 2010 criterion, which has a maximum score of 25—was also considered good.^9^

The main results about the implications of technology in childhood are detailed in Tables 2 and 3.

**Table 2:** Description of articles addressing negative factors related to technology (n=6).

Authors (year)	Media type	Main results
Takeuchi et al. (2018)^3^	Internet	Higher frequency of internet use was associated with decreased verbal intelligence and smaller increases in brain volume after a few years. The areas of the brain affected are related to language processing, attention, memory, and executive, emotional and reward functions.
Slater et al. (2017)^13^	Games (Internet)	Internet games that focus on appearance can be harmful to girls’ body self-image.
Folkvord et al. (2017)^15^	Games (*advergames*)	Advertising games (advergames) encourage the consumption of unhealthy foods.
Slater et al. (2016)^14^	Television	Children are able to absorb or internalize social messages about sexualization, illustrated in the study as the desire for sexualized clothing. Internalizations had a negative impact on their body self-image.
Takeuchi et al. (2016)^11^	Games (*videogames*)	Playing video games for long periods can cause direct or indirect interruption in neural systems’ development, which can be related to an unfavorable neurocognitive development, especially verbal intelligence.
Takeuchi et al. (2015)^12^	Television	Watching television affects the regional volume of the brain associated with verbal language. TV watching time was negatively correlated with verbal intelligence quotient. It can indirectly affect sensorimotor areas.

**Table 3:** Description of articles addressing positive factors related to technology (n=2).

Authors (year)	Media type	Main results
McNeill et al. (2019)^16^	Television, Games, Apps	Use of electronic applications for less than 30 minutes a day and limited media viewing could be associated with cognitive and psychosocial development of preschool-age children.
Yu and Park (2017)^17^	Internet	Use of internet to socialize, exchange ideas and talk about concerns. An opportunity to socialize and make friends.

After reading and analysis, the articles were classified and distributed into two categories according to their approach: negative aspects (n=6) and positive aspects (n=2). The review results are reported below.

### Negative aspects

Six of the studies linked technologies to negative aspects. The papers highlitghed intellectual complications,^3,11,12^ body image dissatisfaction^13,14^ and encouragement of unhealthy food consumption.^15^ Table 2 shows the main information.

Excessive internet use is transversally associated with lower cognitive functioning and reduced volume of several areas of the brain. In longitudinal analyses, a higher frequency of internet use was associated with a decrease in verbal intelligence and a smaller increase in the regional volume of gray/white matter in several brain areas after a few years. These areas relate to language processing, attention and executive functions, emotion and reward.^3^

In a study conducted with 80 British girls aged 8 and 9 years, appearance-focused games led participants to have a greater dissatisfaction with their appearance compared to control girls, who were not exposed to such games. Therefore, internet games that address appearance can be harmful to girls’ body self-image.^13^

It’s not just appearance-focused games that have a negative impact on body image. TV shows, depending on the approach, can also impact negatively psychological development. In a study with Australian girls, some TV shows aimed for the age group of 6-9 years focused on sexualization were absorbed or internalized as social messages by children. The authors stated that the exposure made these girls whish to wear sexualized clothes and create negative relationship with their body image.^14^

Furthermore, a study with 562 Dutch and Spanish children reported that, among Dutch children, games with advertisements (advergames) for high-calorie foods stimulated the consumption of unhealthy foods, while those who played other games with advertisements other that food-related, were less inclined to this eating habit.^15^ Thus, depending on what the child is exposed to, some influences may not be beneficial.

Video games were associated with increased mean diffusivity in cortical and subcortical areas. That is, prolonged video game use was associated with negative consequences, as it can directly or indirectly interrupt the development of neural systems and cause unfavorable neurocognitive development, especially when it comes to verbal intelligence.^11^

Another study on children’s exposure to television, identified a negative effect on the gray matter of the frontal area of the brain with consequences for verbal language. No changes were identified in sensorimotor areas as related to TV watching time; the effect may not be direct, since watching this media is often associated with less physical activity, which, in turn, causes changes in the volume of gray matter in sensorimotor areas.^12^

### Positive aspects

Only two studies brought the positive aspects of technology use, related to cognitive and psychosocial development^16^ and forms of interpersonal relationships.^17^ Main information is shown in Table 3.

Associations of electronic media use with psychosocial development and the executive function among 3- and 5-year-olds, particularly related to total screen time, TV shows viewing, and application use were assessed by the authors, who concluded that cognitive and psychosocial development in children 12 months later was positive when exposure to these media lasted less than 30 minutes a day.^16^

In a study conducted with 2,840 students in South Korea, children with depressed mood were more likely to use the internet to socialize, exchange ideas and talk about their concerns as a way to meet their friendship needs. The Internet can be beneficial for children, who can take advantage of online opportunities for socialization and friendships based on common interests.^17^

## DISCUSSION

The studies analyzed, in general, show that children currently spend a significant amount of time on the Internet or other means of information, and consider that this exposure can have positive and negative impacts on children’s cognitive development and learning skills.

As for the negative impacts of this habit in childhood, the higher frequency of internet use is associated with a significant decrease in verbal intelligence, mainly related to language skills and concentration/attention abilities. One study reported frequent internet use by children as related to decreased memory performance.^18^

Another issue that must be taken into account is the number of games emerging all the time with new elements of fun and entertainment to attract children. An alert should be raised, however, about destructive websites such as the Blue Whale Challenge, which target vulnerable children and young people, threaten their physical integrity and are completely unethical, leading to the gradual destruction of society.^19^

On the other hand, researchers have identified, among the most frequent purposes in allowing children access technology declared by parents, the promotion of problem-solving skills (56.7%), learning of basic mathematics (53.8%), developing hand-eye coordination (46.2%), introduction to reading (51%), language (47.1%) and science (26%), as well as entertainment (56.7%).^20^

Based on the studies selected, we point out an unexpected result for parents: the problematic use of electronic devices at an early age can have children show low levels of openness to experiences, increasing the level of emotional instability, impulsive or other behaviors related to attention. Then, we must reinforce that exposure to media must be carefully pondered by parents and guardians as to avoid media dependence and misuse.

Problematic internet use (PIU) is associated with less openness and agreeableness, as children with higher levels of PIU end up with a deficit in social skills and difficulties in establishing interpersonal relationships, which can lead to being less open and visible, or less friendly externally. It was also found that these children tend to experience negative emotions and use the internet as a means of feeling better about their everyday problems or unpleasant feelings. Relationships were also between problematic video game use and behavior problems, specifically related to thoughts, attention, and aggressive behavior.^21^

In order to bypass the negative effects of inappropriate use of the internet, one cannot ignore, on the one hand, the positive side of these technologies. Technology is extensively available and it is almost impossible to remove it from children’s daily lives.^22^ But the negative effects mentioned during the discussion deserve the same attention, as the authors place parental control and moderation as key factors.^23^ In this sense, there is a directly proportional link between parental participation and attention and a less harmful relationship between children and technologies, especially regarding social factors.^24^

Currently, children spend their lives immersed in the world of digital media, and research has consistently shown the growing, early and diversified use of this media. Children exposed to electronics tend to develop a desire for continued use, creating a potentially harmful cycle. Even more worrisome are the effects of digital media on young children by disrupting parent-child interaction, which is critical to a healthy emotional and cognitive development.^25^

There are potential benefits of digital technology as a tool to enhance early childhood development, creativity and social connection, but it is imperative that parents monitor what their children are consuming and help them learn from it.^26^

A review of the literature about media reported an adverse association between screen-based media consumption and sleep health, mainly due to delays in bedtime and reduced total sleep duration. The underlying mechanisms of these associations include:

- Time replacement, that is, time on screens replacing sleep time and time spent other activities.
- Psychological stimulation based on media content.
- Effects of light emitted by devices on circadian timing, sleep physiology, and alertness.^27^

There is, therefore, and evident need to identify the warning signs of excessive technology use in this age group and define the appropriate limit of daily screen time. Children can make a balanced use of technologies, taking advantage of them without exaggeration, favoring communication and the search for information that is relevant to learning.

It is important to emphasize that pre-judgments about technology-dependent children should be avoided, and knowing their feelings about themselves, as well as the factors that bother them, is important, as well as having a sensitive listening to form a vision of ideal approach in this condition of technology dependence by means of suggested strategies to effectively face these difficulties.^28^

Although this review has important and interesting results, some limitations must be listed. First, there the number of studies identified with the criteria of our work was limited. Also, most of the studies were observational. Therefore, experimental research must be carried out as a means to understand the cause-consequence dynamics between media and their implications for child development. Further studies with larger samples and specific age groups, which would be relevant to increase statistical power, are needed.

The analysis of the articles showed positive and negative factors associated with the use of technologies by children. The main losses caused by technology use in childhood are excessive time connected to the internet, worsening of mental health, and changes in the circadian rhythm. The articles mentioned as negative factors the development of intellectual impairments, including verbal intelligence and attention, emotional instability, internet addiction, binge eating and physiological changes.

The main benefits of the use of technologies by children found were the strengthening of friendships and the possibility of greater social connection. For the preschool age group, there is evidence of improvement in cognitive and psychosocial development. Thus, in order to have technology as an ally for healthy child development, parents and guardians should limit the time of use and control the type of content seen and shared by children.

Currently, preventing internet use is an unrealistic measure, since parents and guardians also make great use of technologies. However, because of the new settings imposed by the COVID-19 pandemic, many services have moved towards digitization, including education and social interaction. Internet use nowadays is a reality for all age groups and makes this study relevant; measures aimed at optimizing its use and reducing risks must, therefore, be adopted. Once again, we emphasize the importance of parents and guardians as moderators and update training of health professionals to better guide them.

Further studies are suggested so the notion of risk-benefit of internet use and its long-term consequences for child development is kept up to date.
